# Novel yeast-based biosensor for environmental monitoring of tebuconazole

**DOI:** 10.1007/s00253-023-12944-z

**Published:** 2024-01-03

**Authors:** Filipa Mendes, Eduarda Miranda, Leslie Amaral, Carla Carvalho, Bruno B. Castro, Maria João Sousa, Susana R. Chaves

**Affiliations:** 1 CBMA – Centre of Molecular and Environmental Biology/ARNET – Aquatic Research Network, Department of Biology, School of Sciences, University of Minho, 4710-057 Braga, Portugal; 2 Present Address: Faculty of Pharmacy, University of Porto, Porto, Portugal; 3 Institute of Science and Innovation for Bio-Sustainability (IB-S), School of Sciences, University of Minho, 4710-057 Braga, Portugal

**Keywords:** Pesticide monitoring, Anti-fungal substances, Ergosterol biosynthesis pathway, Engineered biosensor, Analytical tools

## Abstract

**Abstract:**

Due to increasing demand for high and stable crop production, human populations are highly dependent on pesticide use for growing and storing food. Environmental monitoring of these agrochemicals is therefore of utmost importance, because of their collateral effects on ecosystem and human health. Even though most current-use analytical methods achieve low detection limits, they require procedures that are too complex and costly for routine monitoring. As such, there has been an increased interest in biosensors as alternative or complementary tools to streamline detection and quantification of environmental contaminants. In this work, we developed a biosensor for environmental monitoring of tebuconazole (TEB), a common agrochemical fungicide. For that purpose, we engineered *S. cerevisiae* cells with a reporter gene downstream of specific promoters that are expressed after exposure to TEB and characterized the sensitivity and specificity of this model system. After optimization, we found that this easy-to-use biosensor consistently detects TEB at concentrations above 5 μg L^−1^ and does not respond to realistic environmental concentrations of other tested azoles, suggesting it is specific. We propose the use of this system as a complementary tool in environmental monitoring programs, namely, in high throughput scenarios requiring screening of numerous samples.

**Key points:**

• *A yeast-based biosensor was developed for environmental monitoring of tebuconazole.*

•*The biosensor offers a rapid and easy method for tebuconazole detection* ≥ *5* μ*g L*^*−1*^*.*

•*The biosensor is specific to tebuconazole at environmentally relevant concentrations.*

**Supplementary Information:**

The online version contains supplementary material available at 10.1007/s00253-023-12944-z.

## Introduction

Over the last decades, modern agriculture has been increasingly dependent on pesticides and fertilizers to support the growth of the human population (Bommarco et al. 2013). However, despite their critical role in worldwide agricultural production, these substances can have a negative impact in ecosystem and human health (Sharma et al. 2019). It is therefore of utmost importance to monitor agrochemicals in environmental samples, which is currently undertaken using analytical methods, such as high-performance liquid chromatography, gas chromatography, and mass spectrometry. Most of these are sensitive, efficient, and reliable, but require complex procedures performed by highly trained technicians and considerable sample preparation, aside from the high economic cost (Bhadekar et al. 2011; Jaffrezic-Renault 2001). As such, environmental monitoring programs urge for fast, cost-effective, and disposable systems, with the ability to monitor the increasing number of relevant analytes (Rodriguez-Mozaz et al. 2006).

In recent years, biosensors have demonstrated great potential as an alternative or a complementary tool for detection of environmental contaminants (da Costa Silva et al. 2013; Jarque et al. 2016). The use of biosensors in environmental monitoring programs would present countless advantages, particularly in the simplicity of allowing assessment of pollutants in complex matrices without sample preparation (Ma et al. 2022; Moraskie et al. 2021; Wahid et al. 2023). Whole cell-based biosensors have been developed for different fields such as environmental monitoring (Nourmohammadi et al. 2020; Roda et al. 2011) and pharmacology and drug screening (Hillger et al. 2015; Zager et al. 2010). Among whole cell-based biosensors, the yeast *Saccharomyces cerevisiae* emerges as a particularly interesting model due to its simple genetic manipulation, resilience to harsh conditions, established human safety, and propensity to fast high-throughput functional genomics to identify specific traits (Khurana and Lindquist 2010; Schofield et al. 2007). Biosensors based on this fungal model have already been used to detect endocrine disruptive substances (Cevenini et al. 2018; Lobsiger et al. 2019; Sanseverino et al. 2005). Nonetheless, despite its potential and the invaluable add-on to the available analytical toolbox, yeast-based biosensing is underexplored, probably due to limited utility of detection of only a compound class (Nartin-Yken 2020). Expanding the range of compounds detected by yeast-based biosensors would therefore allow for much needed streamlined simultaneous monitoring of various types of environmental contaminants.

Fungicides are among the most frequently used pesticides and are essential to control diseases that reduce crop production and threaten food security (Zubrod et al. 2019). As a consequence of their common and extensive use, fungicides reach aquatic ecosystems and have been detected at higher concentrations than herbicides and insecticides in European surface waters (Stehle and Schulz 2015). Despite current efforts towards a more sustainable use of pesticides, fungicide use is predicted to increase as a result of increased fungal resistance, invasive species, and climate change (Zubrod et al. 2019). As such, high-throughput detection technologies of these substances in water samples constitute a useful add-on for environmental scientists and managers, and fungi-based biosensors are particularly suitable for the job.

Among the most common classes of fungicides are demethylation inhibitors (a group of sterol biosynthesis inhibitors), which include azoles. They are regularly used to eliminate or control diseases caused by fungi and oomycetes in agricultural crops and as therapeutic drugs for medical purposes (Price et al. 2015). Tebuconazole (TEB) is one of the most frequently applied (Azevedo et al. 2015), and it is thus often detected in aquatic ecosystems, where there are concerns about its potentially negative effects on non-target aquatic biota at environmentally-relevant concentrations (Cuco et al. 2020; Pimentão et al. 2020; Zubrod et al. 2015). Like other azoles, TEB has a well-known specific mode of action, inhibiting the activity of lanosterol 14-α demethylase, a cytochrome P450 enzyme encoded by the *ERG11* gene (CYP51). This affects ergosterol biosynthesis, ultimately leading to accumulation of sterol precursors and ergosterol depletion (Joseph-Horne et al. 1995; Lamb et al. 1999). Ergosterol is the major component of fungal membranes, regulating membrane integrity, fluidity, and permeability; consequently, alterations in ergosterol homeostasis and limitations in its availability can affect and even prevent fungal growth (Kathiravan et al. 2012; Kodedová and Sychrová 2015). Ergosterol biosynthesis is a highly conserved and complex process that is regulated by a cascade of 25 biosynthetic enzymes, coded by *ERG* genes (Jordá and Puig 2020). In *S. cerevisiae*, this pathway is mainly regulated by the transcription factor Upc2p, which undergoes a conformational change when ergosterol levels decrease, triggering Upc2p translocation to the nucleus and binding to *ERG* promoters, thus causing transcriptional induction of most *ERG* genes (Marie et al. 2008; Yang et al. 2015).

Bearing this in mind, the goal of this work was to develop a novel yeast-based biosensor for environmental monitoring of TEB in aqueous samples. For that purpose, we used a modified yeast strain expressing a reporter gene downstream of specific promoters that are expressed after exposure to TEB. Moreover, we aimed to clarify if the biosensor developed was specific to TEB or if it was also capable of detecting other azole compounds, since most of these fungicides share the mode of action (Joseph-Horne et al. 1995; Lamb et al. 1999). For this purpose, we selected myclobutanil (MYC), another triazole fungicide used to control fungal diseases in crop production (Fonseca et al. 2019), as well as antifungal pharmaceutical azoles used to treat fungal infections, which share the mode of action but reach aquatic environments via wastewater (Berkow and Lockhart 2017; Crowley and Gallagher 2014), namely clotrimazole (CLO) and fluconazole (FLU).

## Materials and methods

### Yeast strains and plasmids

All *S. cerevisiae* strains and plasmids used in this study are listed in the supplementary material (Table S1). *S. cerevisiae* BY4741 was used throughout this study as the wild-type strain. Due to the small size (19 kDa), the absence of post-translational modifications, and its high sensitivity, the yeast codon-optimized version of NanoLuciferase [yNLuc (Masser et al. 2016)] was selected as the reporter gene.

The sequences encoding the promoters of *ERG3*, *ERG6*, *ERG11*, and *ERG*25 genes were amplified by polymerase chain reaction (PCR) using genomic DNA isolated from BY4741 cells. Fusions of these promoters upstream of yNLuc were generated by overlap extension PCR and then cloned into pRS426 (Sikorski and Hieter 1989) by gap repair. Primers used in this study are listed in the supplementary Table S2. After confirmation by Sanger sequencing, the wild-type strain BY4741 was transformed with plasmids using the LiAc/SS Carrier DNA/PEG method (Gietz and Woods 2006).

### Growth conditions and treatments

Strains were grown in synthetic complete glucose medium (SC-glucose; 2% (w/v) glucose; 0.17% (w/v) yeast nitrogen base without amino acids and without ammonium sulfate; 0.1% (w/v) L-proline, 0.14% (w/v) drop-out mixture lacking leucine, histidine, tryptophan, and uracil; 0.04% (w/v) leucine; 0.008% (w/v) tryptophan; 0.008% (w/v) histidine; and 0.008% (w/v) uracil) at 30 °C in an orbital shaker at 200 rpm, with a ratio of flask volume/medium of 5:1. Strains transformed with the indicated plasmids were grown in the same medium lacking uracil. Solid medium contained 2% (w/v) agar and 0.5% (w/v) ammonium sulfate instead of L-proline. L-proline was used as the nitrogen source in liquid assays since previous studies demonstrate that a medium containing L-proline as the only source of nitrogen increases the sensitivity of the wild-type strain S288c to the antifungal FLU (Stella et al. 1998).

For all assays, cells were grown overnight and then diluted in fresh medium to OD_640 nm_ = 0.1 and grown for an additional 3 h. Afterwards, cells were treated with TEB (0–5000 μg L^−1^) or MYC, CLO, or FLU[(0–162 nM (molar equivalent of 50 μg L^−1^ of TEB)] from Sigma Aldrich and/or the equivalent volume of DMSO as a negative control (lower than 0.5%) for up to 30 h. Samples were taken at different time points for subsequent assays. Samples of the wild-type strain BY4741 were collected for cell growth and survival assays, assessment of plasma membrane integrity, evaluation of lipid raft distribution, and quantitative real-time PCR (qRT-PCR). *S. cerevisiae* BY4741 cells harboring p*ERG3*pryNluc (*ERG3*pr), p*ERG6*pryNluc (*ERG6*pr), p*ERG11*pryNluc (*ERG11*pr), or p*ERG25*pryNluc (*ERG25*pr) were used for bioluminescence assays.

### Cell survival assays

Cell survival of BY4741 cells treated in the absence or presence of 500 and 5000 μg L^−1^ of TEB was evaluated by counting of colony forming units (c.f.u.). Briefly, five 10-fold serial dilutions of the cultures were performed, and 40 μL of 10^−4^ or 10^−5^ dilutions (according to each condition) were plated onto yeast extract-peptone-dextrose plates (YEPD, 1% (w/v) yeast extract, 2% (w/v) peptone, 2% (w/v) glucose, and 2% (w/v) agar) and incubated at 30 °C for 48 h. The percentage of cell survival was calculated from the number of c.f.u. of each condition in relation to time zero and the control without any treatment.

### Assessment of plasma membrane integrity

To assess loss of plasma membrane integrity, usually associated with a cell death process (Kroemer et al. 2009), cells were stained with the cell-impermeant dye propidium iodide (PI, Sigma Aldrich). PI was added to yeast cell suspensions to a final concentration of 2000 μg L^−1^ followed by incubation at room temperature for 10 min. Plasma membrane integrity of BY4741 cells treated in the absence or presence of 500 and 5000 μg L^−1^ of TEB was assessed at 0, 3, and 6 h of treatment. After 6 h of treatment, cells were harvested, washed, transferred to fresh medium without fungicide, and grown for an additional 3 h; then, plasma membrane integrity was re-assessed.

Detection of fluorescence was performed with a CytoFLEX (Beckman Coulter Inc.) flow cytometer. Cells with red fluorescence were considered to have lost their plasma membrane integrity. Data was analyzed using CytExpert software.

### Evaluation of lipid raft distribution by filipin staining

The localization of lipid rafts, plasma membrane nanodomains containing ergosterol in association with sphingolipids (Wachtler and Balasubramanian 2006), was inferred by fluorescence microscopy (Leica Microsystems DM-5000B epifluorescence microscope) using filipin staining (Filipin III from *Streptomyces filipinensis*, Sigma Aldrich). Filipin is a naturally fluorescent antibiotic dye that binds to ergosterol but not to esterified sterols, and is thus a broadly accepted method to detect regions with high sterol content in the plasma membrane of fungal species (Maxfield and Wüstner 2012). Briefly, BY4741 cells were treated for 6 h in the absence or presence of 500 μg L^−1^ TEB and then collected at an OD_640 nm_ = 0.5 and concentrated 20× in sterile water. Immediately before visualization, cells were incubated with 0.1 g L^−1^ filipin from a stock solution of 5 g L^−1^ (w/v) in methanol for 1 min in the dark (Pacheco et al. 2013; Santos-Pereira et al. 2021). Cells were then mounted on slides with the anti-fading agent Vectashield (Vector Laboratories) to overcome the instability of this dye and visualized in an epifluorescence microscope.

### qRT-PCR

The effect of TEB on the expression levels of *ERG10*, *ERG7*, *ERG11*, *ERG25*, *ERG6*, and *ERG3* genes was assessed by qRT-PCR. Total RNA from cells exposed to 0, 5, or 50 μg L^−1^ TEB for 8 h was extracted using TRIzol/chloroform and RNA Clean and Concentrator-5 Kit (Zymo Research). Briefly, the upper aqueous phase from TRIzol/chloroform extraction was transferred to a new tube, and RNA was purified with the RNA Clean and Concentrator-5 Kit according to manufacturer’s instructions. Then, cDNA was synthesized by reverse transcription from 500 ng of total RNA according to the manufacturer’s instructions (iScript™ cDNA Synthesis Kit, Bio-Rad Laboratories). Real-time PCR reactions were performed in a CFX96^TM^ Real-Time System C100^TM^ Thermal Cycler (Bio-Rad Laboratories) using the KAPA SYBR® FAST qPCR Master Mix (2×) Kit (Sigma-Aldrich) according to manufacturer’s instructions. PCR controls with no template were also performed for each primer pair. Three biological replicates were performed, and each sample was measured in duplicate. Quantification was performed using the 2^-ΔΔCt^ method (Livak and Schmittgen 2001), and expression of *ERG* genes was normalized to A*CT1*.

### Evaluation of biosensor performance in environmental water samples

To test the performance of the biosensor in real samples, environmental water samples were collected and tested. To maintain aseptic conditions, environmental water samples and distilled water were filtered prior to analyses. Briefly, *ERG25*pr cells were grown overnight, then diluted in fresh medium to OD_640 nm_ = 0.1, and grown for an additional 3 h. Afterwards, cells were collected and resuspended in medium containing 9 parts distilled water (control of the experiment) or environmental water samples and 1 part 10× concentrated SC-glucose medium. Samples spiked with 10 μg L^−1^ of TEB and/or the equivalent volume of DMSO and without supplementation were used for bioluminescence assays.

### Bioluminescence measurements

For bioluminescence assays, 1 mL of culture (OD_640 nm_ = 0.5) was collected and resuspended in SC-Glucose medium (pH 8) containing 6.25 mM PEG 3350. Nano-Glo substrate (Promega GmbH, Germany) was diluted 1:100 with the supplied buffer and mixed 1:4000 with whole cells in white opaque 96-well microplates (OptiPlate-96). Bioluminescence emissions were recorded using a Varioskan Flash multimode reader (1000 ms integration time). Light emission was expressed in arbitrary units (a.u.).

### Statistical analysis

A one- or two-way analysis of variance (ANOVA) was used to test the effect of fungicide (in most cases TEB) concentrations on yeast growth, viability, plasma membrane integrity, gene expression, or bioluminescence signal. A Dunnett test was used to assess differences relatively to the control. A significance level of 0.05 was employed in all analyses.

## Results

### TEB induces transcription of *ERG* genes at concentrations that do not affect growth, viability, and ergosterol distribution

**Fig. 1:**
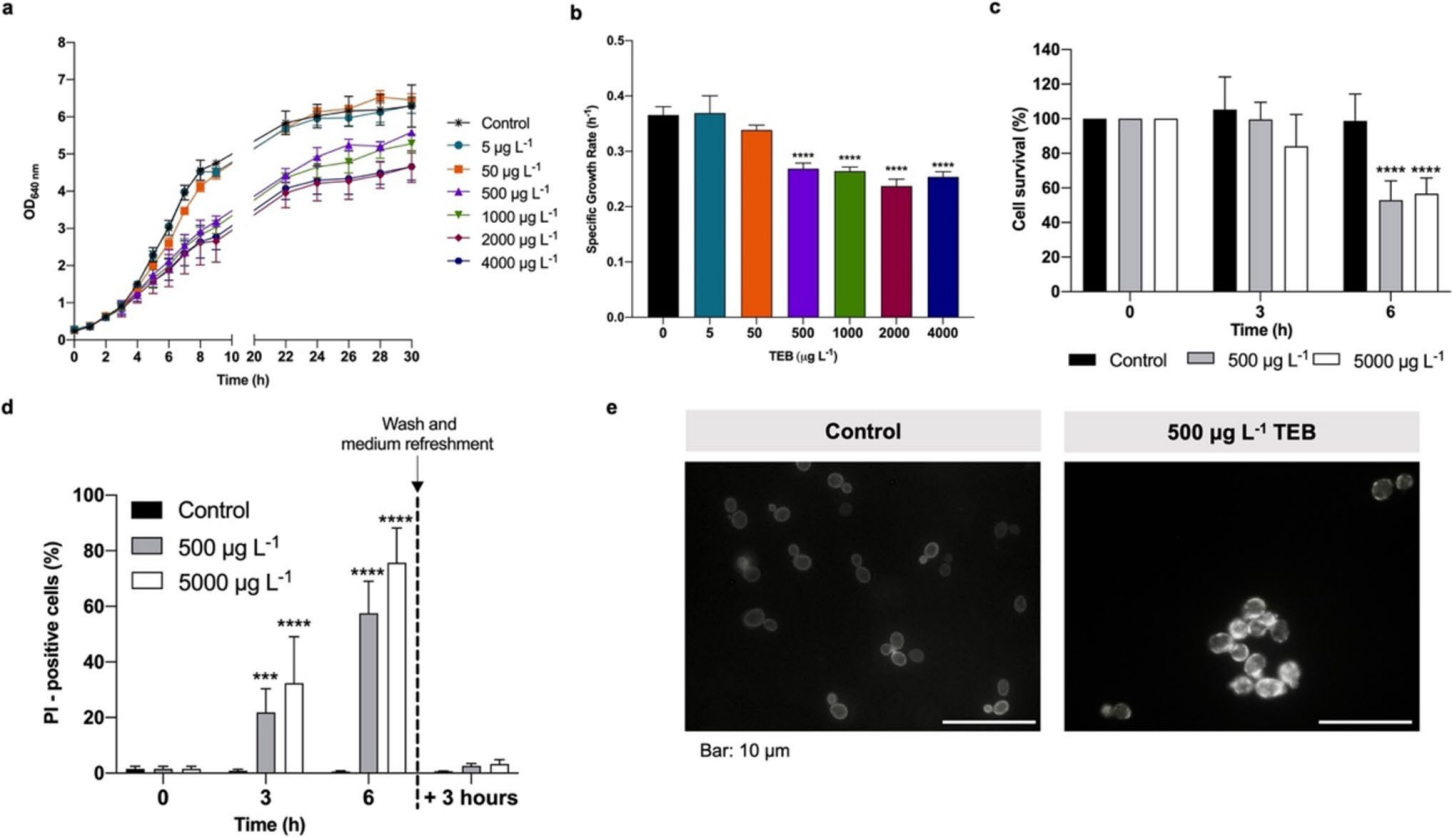
Effect of high concentrations of TEB on growth, cell survival, plasma membrane integrity, and lipid raft distribution of *S. cerevisiae* BY4741 cells. Cells were grown overnight and diluted to an OD_640 nm_ = 0.1, transferred to fresh medium and grown for an additional 3 h; then, cells were treated with TEB. **a** OD_640 nm_ of cells grown in the absence or presence of increasing concentrations of TEB (5–4000 μg L^−*1*^) were registered over time, and the specific growth rates **(b)** were calculated considering the exponential phase in **(a)**. **c** Cell survival of BY4741 cells treated with 0, 500, or 5000 μg L^−1^ TEB was determined by standard dilution plate counts and expressed as a percentage of c.f.u. on YEPD plates in relation to time 0. **d** Plasma membrane integrity was determined by PI staining of cells treated with 0, 500, or 5000 μg L^−1^ TEB at time points 0, 3, and 6 h and after 3 h of washing cells and medium refreshment. **e** For lipid raft visualization, cells were treated for 6 h in the absence (control) or presence 500 μg L^−1^ of TEB; then, cells were collected and stained in the dark with 0.1 g L^−1^ filipin, immediately before visualization under the microscope. Representative fluorescence images of each condition are shown. Due to fast photobleaching of filipin, fluorescence intensity is not associated with ergosterol levels and only localization can be inferred. The data displayed are the mean ± standard deviation of three independent experiments. Asterisks (***P ≤ 0.001; ****P ≤ 0.0001) depict significant differences relative to the control

To characterize the effect of TEB in *S. cerevisiae*, we first assessed the growth of laboratory strain BY4741 in the presence of increasing concentrations of this fungicide for up to 30 h. We observed that medium to low TEB concentrations (50 and 5 μg L^−1^) did not affect the growth of BY4741 cells (Fig. 1a, b), while exposure to high concentrations (500 to 4000 μg L^−1^) for 6 h or longer inhibited cell growth (Fig. 1a), leading to a decrease in the specific growth rate and in the final biomass (Fig. 1b). Nonetheless, cells still grew, suggesting that those concentrations can limit growth of yeast cells but do not result in high levels of cell death. Indeed, we found that exposure to 500 or 5000 μg L^−1^ of TEB for 3 h did not affect cell survival and after 6 h cell survival decreased only slightly, for both concentrations tested (Fig. 1c). As shown in Fig. 1d, exposure to 500 and 5000 μg L^−1^ of TEB led to a significant increase in PI staining after 3 h and 6 h, indicating that plasma membrane integrity is compromised. However, this effect was reversible, since the percentage of cells staining with PI drastically decreased if cells were washed after exposure to TEB and subsequently incubated in fresh medium for 3 h (Fig. 1d). We further assessed the effect of TEB on the plasma membrane by visualizing distribution of lipid rafts in control and TEB-treated cells (Fig. 1e). We observed the characteristic punctuated pattern of filipin staining at the plasma membrane in control cells, but intracellular spots became apparent after 6 h of exposure to 500 μg L^−1^ TEB, indicating that TEB-treated cells exhibit intracellular accumulation of ergosterol. A similar phenotype was observed for the highest concentration (50, 000 μg L^−1^, data not shown).

We further found that even higher concentrations (50,000 μg L^−1^) and longer treatment periods (up to 72 h) did not increase mutation frequencies of *S. cerevisiae* cells (supplementary material Table S3). This indicates that TEB is not genotoxic and thus a biosensor would not be prone to TEB-induced mutations.

**Fig. 2:**
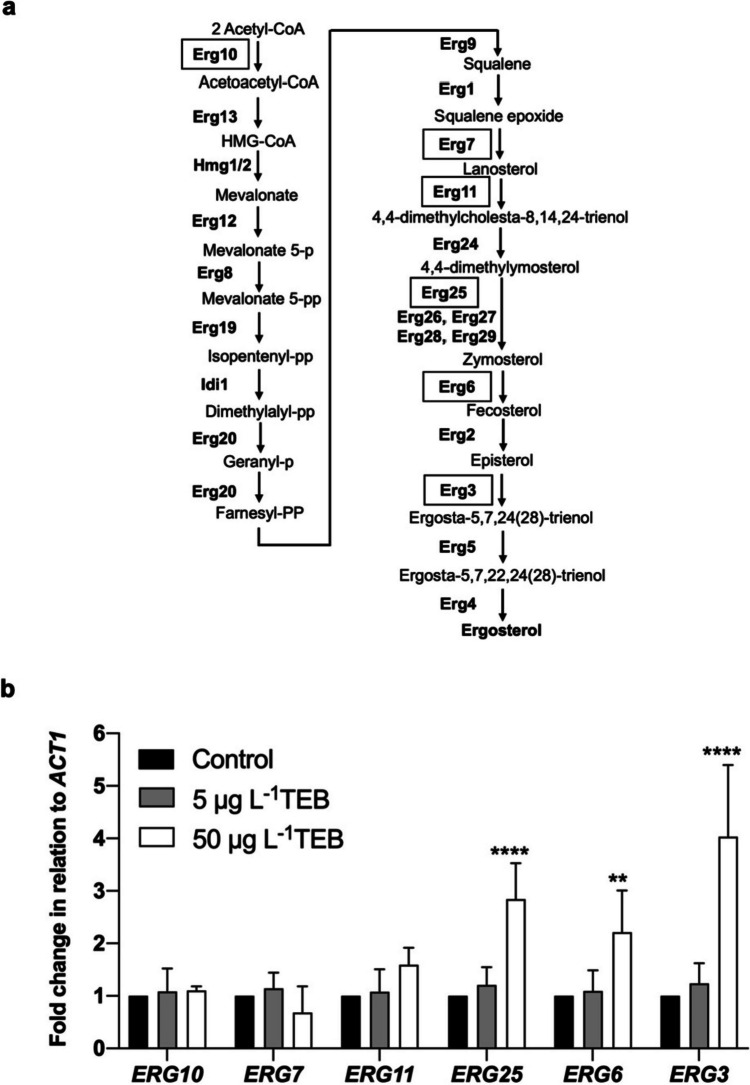
Effect of low TEB concentrations on genes involved in the ergosterol biosynthetic pathway of *S. cerevisiae*. **a** Ergosterol biosynthetic pathway in *S. cerevisiae*. The levels of boxed genes (*ERG10*, *ERG7*, *ERG11*, *ERG25*, *ERG6*, and *ERG3*) were assessed by qRT-PCR. **b** *S. cerevisiae* BY4741 wild-type cells were treated for 8 h in the absence (control) or presence of 5 and 50 μg L^−1^ of TEB; The *ACT1* gene was used as the normalizer. Expression values are represented as the fold change relative to the untreated cells (control) and correspond to the mean ± standard deviation of three independent experiments. Asterisks (**P ≤ 0.01; ****P ≤ 0.0001) depict significant differences relative to the control

Next, we characterized the effect of TEB on the expression levels of *ERG* genes involved in different steps of the ergosterol biosynthesis pathway (Fig. 2a). We found that a low concentration (50 μg L^−1^) leads to upregulation of several genes involved in late steps, although no changes were observed in response to 5 μg L^−1^. As shown in Fig. 2b, 50 μg L^−1^ of TEB increased the relative expression of *ERG6*, *ERG25*, and *ERG3* two, three, and four-fold, respectively, when compared to non-treated cells (control). In contrast, the relative expression of *ERG10* and *ERG7* was not significantly altered by either concentration. Although *ERG11* transcription also tended to increase, this change was not statistically significant (Fig. 2b). Based on these results, *ERG11*, *ERG25*, *ERG6*, and *ERG3* promoters were chosen to construct yeast-based biosensors.

### A novel biosensor design based on yNLuc expression from *ERG* promoters

**Fig. 3:**
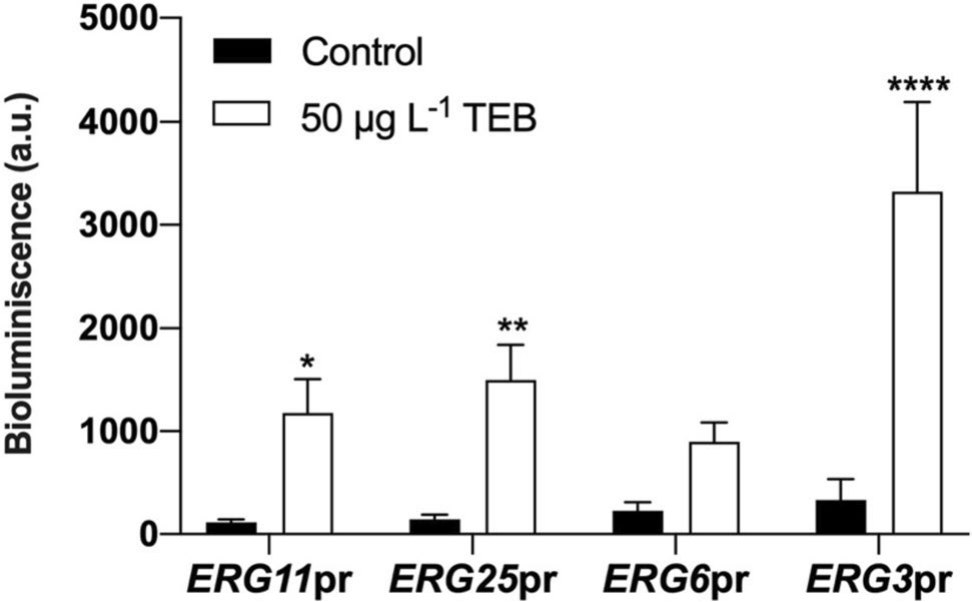
Response of biosensor after exposure to TEB. *ERG11*pr, *ERG25*pr, *ERG6*pr, and *ERG3*pr cells were incubated in the absence (control) or presence of TEB (50 μg L^−1^) for 8 h, cells were collected, and bioluminescence was assessed. Values are mean ± standard deviation of three independent experiments. Asterisks (*P ≤ 0.05; **P ≤ 0.01; ****P ≤ 0.0001) depict significant differences relative to the control

We found that biosensors obtained by genetically engineering *S. cerevisiae* cells with a vector expressing yNluc downstream of *ERG11*, *ERG25*, *ERG6*, or *ERG3* promoters produced an increased bioluminescence signal in TEB-treated cells, but the signal was only significantly different from control cells in *ERG11*pr, *ERG25*pr, and *ERG3*pr biosensors (Fig. 3). Since *ERG*11 is a direct target of TEB (Lamb et al. 1999) and *ERG25*pr and *ERG3*pr biosensors produced a bioluminescence signal with higher significance, we chose only the latter two for further analysis.

**Fig. 4:**
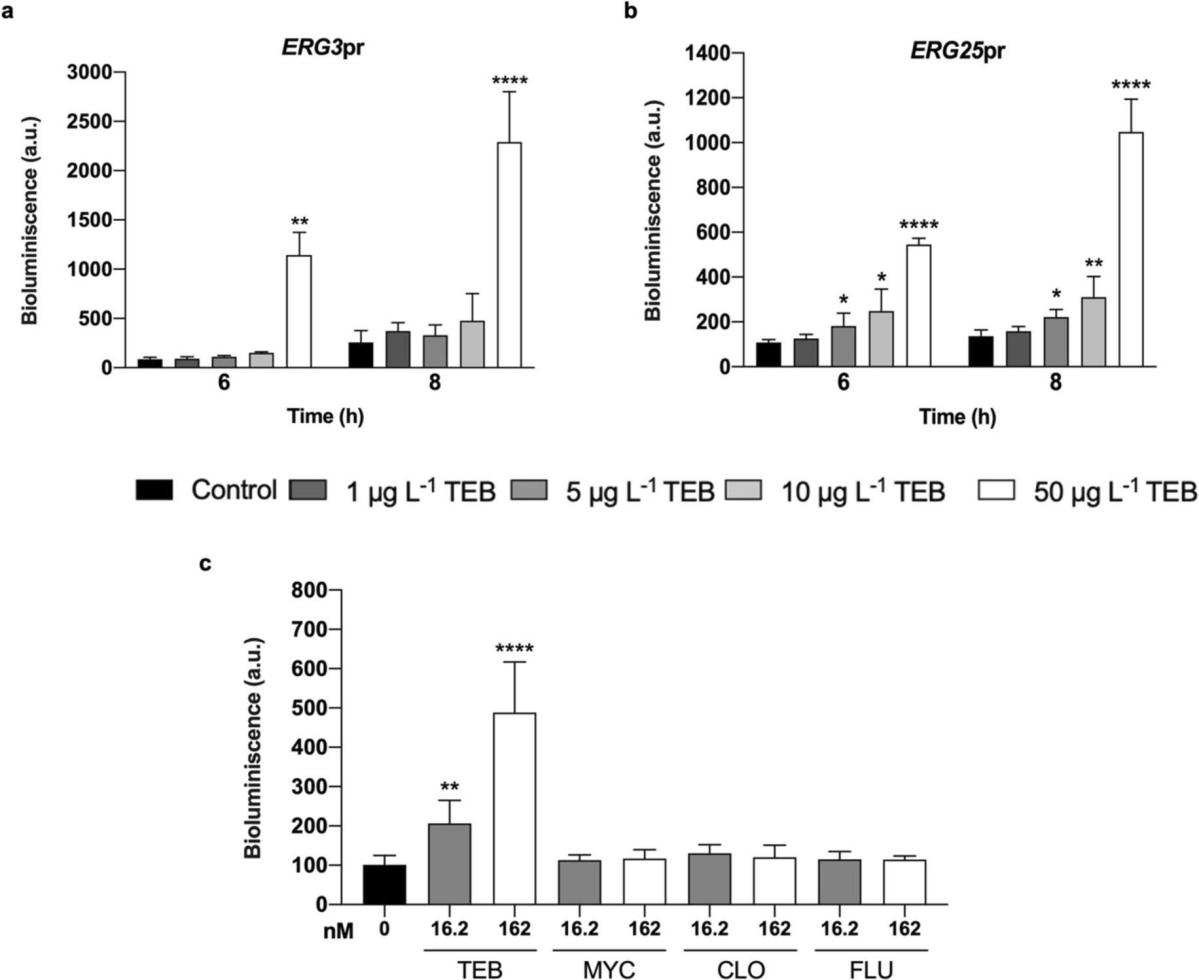
Biosensor sensitivity and selectivity to TEB. Sensitivity of biosensors **a** *ERG3*pr and **b** *ERG25*pr was determined after incubation in the absence (control) or presence of increasing concentrations of TEB (1–50 μg L^−1^). Afterwards, bioluminescence was recorded after 6 and 8 h of treatment. Estimated values of *ERG25*pr LoD [3.3x (σ / S)] and LoQ [10x (σ / S)] based on this experiment were: LoD = 0.84/1.24 and LoQ = 2.55/3.77 (after 6/8 h), consistent with the statistically significant sensitivity of the biosensor of 5 μg L^−1^. **c** For determination of *ERG25*pr selectivity, *ERG25*pr cells were exposed to 0, 16.2, or 162 nM (5 and 50 μg L^–1^, respectively) of TEB, MYC, CLO, and FLU for 6 h, after which bioluminescence was assessed. Values are mean ± SD of three independent experiments. Asterisks (*P ≤ 0.05; **P ≤ 0.01; ****P ≤ 0.0001) depict significant differences relative to the control

Upon further analysis, we observed that the *ERG3*pr biosensor only detected the higher concentration of TEB (50 μg L^−1^), and lower concentrations resulted in a bioluminescence level comparable to untreated cells (Fig. 4a). On the other hand, the *ERG25*pr biosensor detected two lower concentrations, 5 and 10 μg L^−1^, after both 6 h and 8 h of exposure (Fig. 4b). We further verified that 4 h of exposure to 50 and even 100 μg L^−1^ was not sufficient for a significant response in this biosensor, whereas extending the time to 24 h only slightly increased the TEB-induced bioluminescence signal, while also increasing in the control (not shown).

In further analysis of the performance of the *ERG25*pr biosensor, we found there was a significant bioluminescence signal in response to 5 μg L^−1^ and 50 μg L^−1^ of TEB, which was not observed in response to equivalent molar concentrations (16.2 nM and 162 nM) of the azoles MYC, CLO, or FLU (Fig. 4c). Since our final goal is to use the biosensor developed to detect TEB in environmental water samples, we further tested the performance of the biosensor in a wide range of samples collected from eight different sources and hence with different composition (analysis provided in supplementary material Table S4).

**Fig. 5:**
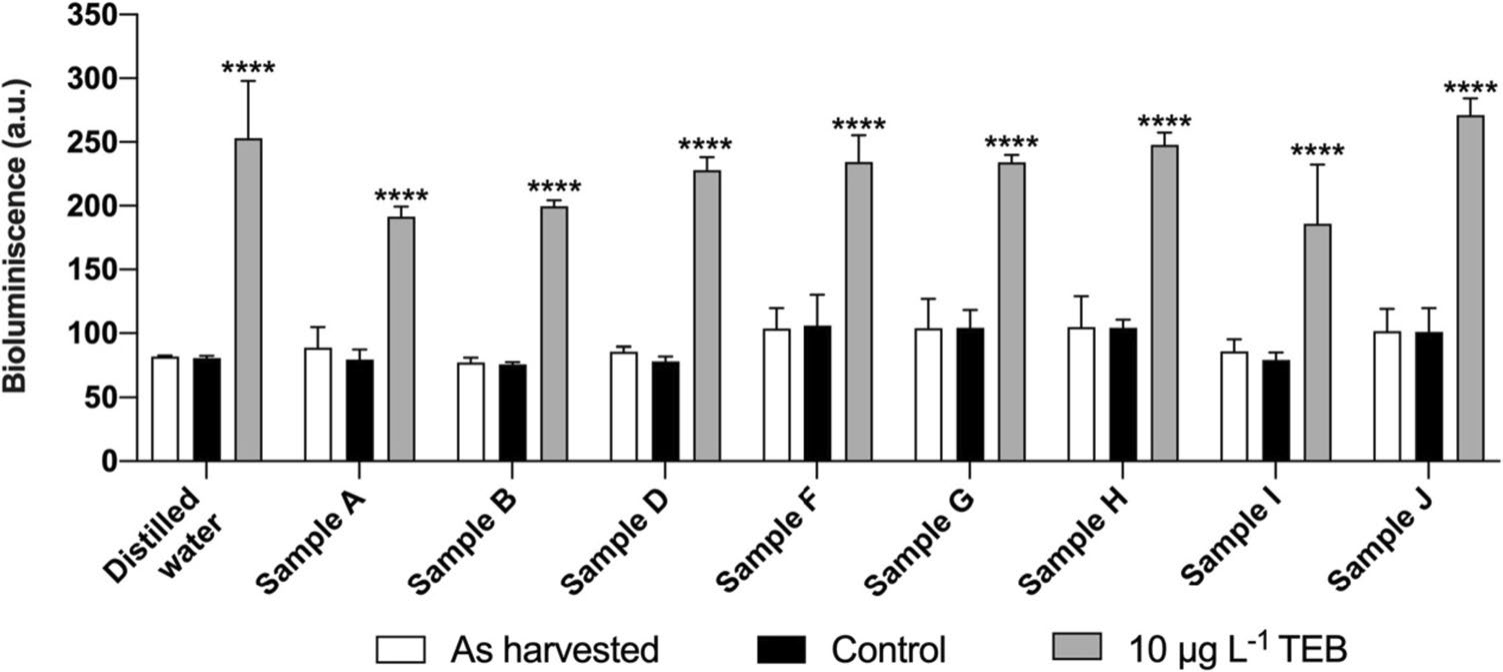
Proof-of-concept of biosensor functionality. Eight environmental water samples were analyzed using biosensor *ERG25*pr. Samples (A, B, D, F, G, H, I, and J) were collected and filtered. *ERG25*pr cells were incubated in 10*×* concentrated medium diluted in water samples as harvested, without TEB supplementation (white), in the presence of the equivalent volume of DMSO control (black), and spiked with 10 μg L^−1^ of TEB (gray), for 6 h. Afterwards, bioluminescence was recorded. Results were obtained in 1 working day. Values are mean ± SD of two independent experiments. Asterisks (****P ≤ 0.0001) depict significant differences relative to the control and as harvested

As a proof-of-concept, the environmental water samples collected were analyzed using the *ERG25*pr biosensor, spiked with 10 μg L^−1^ of TEB or the equivalent volume of DMSO, with distilled water used as a control. As seen in Fig. 5, the bioluminescence signal obtained was very similar between distilled water and the collected samples, and the *ERG25*pr biosensor was able to detect TEB in all spiked samples. We also observed that DMSO (solvent control) did not significantly affect the basal bioluminescence signal (Fig. 5-white bars). Environmental water samples were also analyzed by analytical methods, and results showed that TEB was only detected at vestigial concentrations in sample A (0.014 μg L^−1^) and not detected in the remaining samples, meaning its concentration was below the detection limit (LoD = 0.005 μg L^−1^) (supplementary material Table S5). Other fungicides such as carbendazim, dimethomorph, and metalaxyl were also detected at vestigial concentrations, while copper was detected in all samples (supplementary material Table S5). This analysis also revealed the presence of other polluting substances revealing human presence, namely metformin, a drug used to treat type 2 diabetes that was detected in samples I and J.

## Discussion

TEB is widely used in agriculture and viticulture to control a plethora of fungal diseases (Baćmaga et al. 2022). We found no specific information about the regulatory limits of TEB; in Europe, it is not listed as a priority substance and thus no environmental quality standards have been defined. However, the Groundwater Directive from the European Union Commission determines that the concentration of a single pesticide in groundwater should not exceed 0.1 μg L^-1^ (Directive 2006/118/EC 2006). Locally, TEB has been included in the list of pesticides to monitor to assess the quality of drinking water in Portugal, although specific regulatory limits have not yet been imposed (Decree-Law no 306/2017 of 27.08.2017 2017; Decree-Law no 69/2023 of 21.08.2023 2023). In water courses, TEB is frequently detected at concentrations ranging from 1 to 30 ng L^−1^ (Kahle et al. 2008), but can reach 175–200 μg L^−1^ in extreme runoff events (Elsaesser and Schulz 2008; Zubrod et al. 2010). At these concentrations, TEB effects can be potentially high, given its effectiveness against many fungi (Cuco et al. 2020; Pimentão et al. 2020; Zubrod et al. 2015) and that it can also inhibit human cytochrome P450 sterol 14α-demethylases (CYP51) (Warrilow et al. 2013).

Detection of TEB in aqueous samples, such as water, is normally performed by liquid chromatography-tandem mass spectrometry and gas chromatography-mass spectrometry (Huang et al. 2010). Although these methods are reliable and extremely sensitive, with low detection limits (ng L^−1^), they are time-consuming, expensive, and do not conform to green chemistry practices, as they require sample preparation with solvents (Bhadekar et al. 2011; Daverey et al. 2019). Moreover, the necessity of trained technicians and complex procedures prevents implementation in low-tech local laboratories. Further increasing the environmental cost is the carbon footprint associated with shipping of the collection containers and then returning them with the samples. Therefore, it is of utmost importance to strengthen and simplify the environmental monitoring programs available for its detection in aqueous samples. We therefore set out to contribute to monitoring efforts by constructing a yeast biosensor that can detect TEB. For that purpose, it is necessary that environmentally relevant concentrations (below 200 μg L^−1^) (Elsaesser and Schulz 2008; Zubrod et al. 2010) elicit a biological response without negatively affecting cell viability.

As previously shown for other azole compounds (Parker et al. 2008; Pfaller et al. 1997), we found that high concentrations of TEB inhibit growth of *S. cerevisiae* cells and result in a small loss of viability. We further found that exposure to TEB for 3 or 6 h resulted in loss of plasma membrane integrity, a common feature of cell death (Kroemer et al. 2009). Due to TEB effects on ergosterol biosynthesis (FRAC 2022) and since cell viability was only slightly affected and only after 6 h, we hypothesized that this could be the result of a reversible increase in the permeability to the dye and not to plasma membrane disruption. Indeed, washing the cells after exposure to TEB resulted in a substantial decrease in the percentage of cells stained with PI. This indicates that TEB-treated cells did not reach the point of no return in the cell death process, but instead displayed reversible increased permeability to small molecules. A similar phenotype, with alterations in plasma membrane rigidity, has been described for *S. cerevisiae* mutants defective in the last five steps of the ergosterol biosynthesis pathway, particularly *erg2*Δ and *erg6*Δ. In those mutants, accumulation of ergosterol precursors does not allow a tight packing of the lipid bilayer and thus the occurrence of voids in the plasma membrane increases, increasing the sensitivity of these mutants to small molecules (Abe and Hiraki 2009; Emter et al. 2002; Welihinda et al. 1994). In accordance with alterations in plasma membrane integrity, TEB also disturbed lipid rafts, plasma membrane nanodomains enriched in ergosterol and sphingolipids (Wachtler and Balasubramanian 2006), mainly through intracellular accumulation of ergosterol. This pattern in membrane perturbation has been described for methyl-β-cyclodextrin, a sterol chelator that totally extracts ergosterol from plasma membranes, leading to its intracellular accumulation (Baumann et al. 2005; Santos-Pereira et al. 2021). Nevertheless, TEB-treated cells still preserved ergosterol staining at the plasma membrane, suggesting that TEB does not lead to full depletion of ergosterol from plasma membranes but does disturb ergosterol-rich lipid rafts. Consistent with these results, we found that TEB induced expression of several *ERG* genes involved in the late steps of ergosterol biosynthesis. Azole-dependent upregulation of genes involved in the late steps of ergosterol biosynthesis has been demonstrated in *Candida* species (Henry et al. 2000; Song et al. 2003) and in *S. cerevisiae* (Bammert and Fostel 2000; Henry et al. 2002; Kagan et al. 2005), mainly using high concentrations. Here, we demonstrate that a low TEB concentration also upregulates *ERG* genes. Taken together, our results indicate that high concentrations (500–5000 μg L^−1^) of TEB affect growth, viability, and ergosterol distribution of yeast cells, while a lower concentration (50 μg L^−1^) can stimulate a biological response without affecting yeast growth. We concluded that *S. cerevisiae* is both robust and sensitive to develop a novel yeast-based biosensor for TEB detection in environmental water samples.

To construct the biosensor, we genetically engineered *S. cerevisiae* cells with a plasmid expressing yNluc downstream of *ERG11*, *ERG25*, *ERG6*, or *ERG3* promoter. Among these, the *ERG25*pr biosensor was the most sensitive, detecting the presence of TEB in a statistically significant manner at concentrations above 5 μg L^−1^. This *ERG25*pr biosensor also seems to specifically respond to TEB, as it was unable to detect other azoles at low realistic environmental concentrations. We further demonstrate that the biosensor developed can be used in a simple assay, avoiding preparation of environmental water samples, since biosensor performance was not affected by the presence of potential interfering factors such as electrical conductivity (EC), total dissolved solids (TDS), pH, and other substances.

**Fig. 6:**
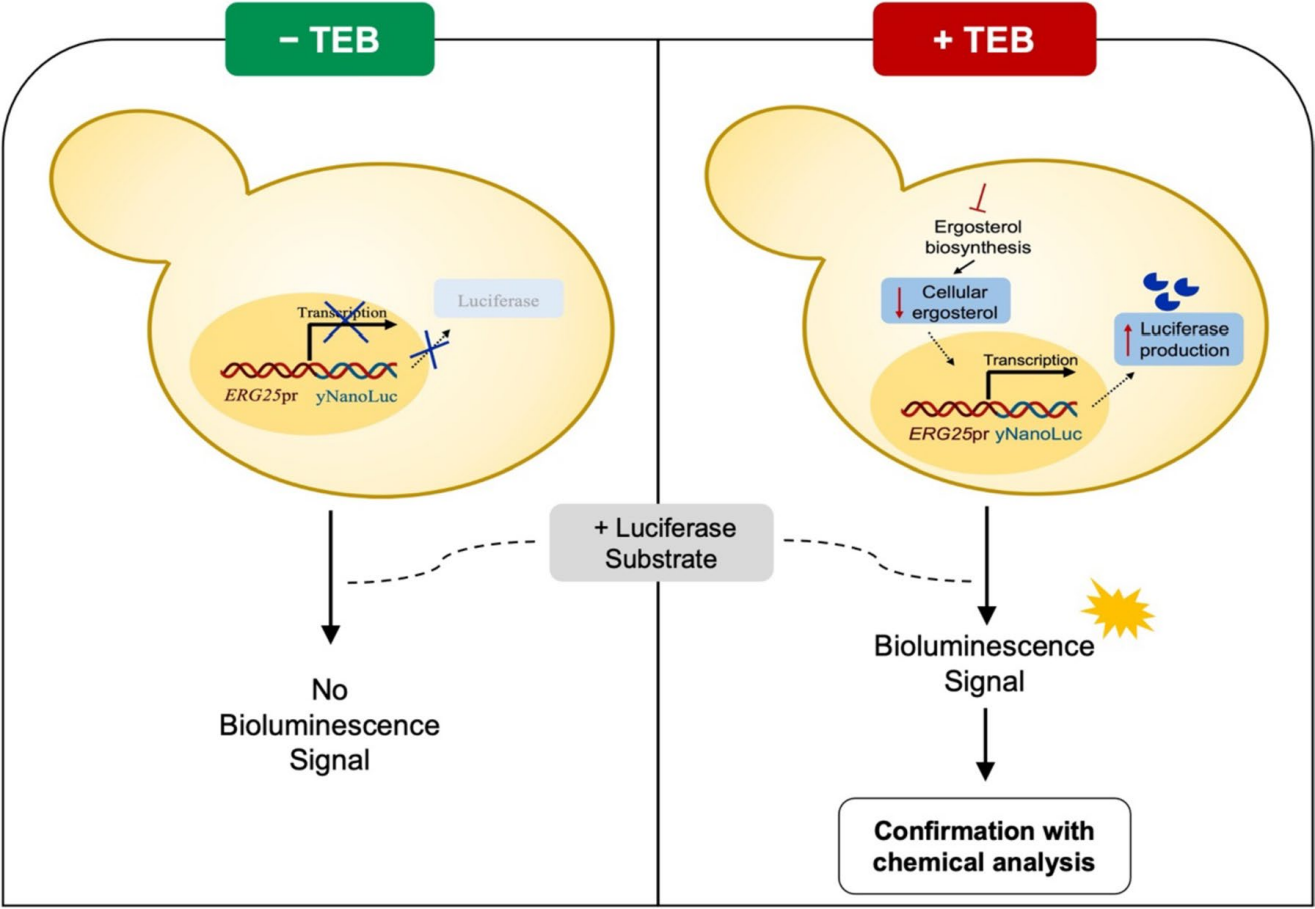
Schematic representation of the novel reporter strain for TEB detection in environmental water samples. Samples from aquatic environments prone to TEB contamination (agricultural fields, rivers, or wastewater management (WWM) effluents) can trigger a cellular response such as inhibition of ergosterol biosynthesis that will lead to the decrease of the cellular levels of ergosterol. Consequently, upregulation of *ERG* genes, particularly *ERG25*, will result in the expression of the reporter gene yNluc. As a consequence, luciferase production will increase and, upon addition of the luciferase substrate, bioluminescence emissions are obtained. Samples without a significative bioluminescence signal indicate that TEB is not present in concentrations above 5 *μ*g L^−*1*^ (left), while samples with a significative bioluminescence signal indicate TEB is present (right) and should be further analyzed by conventional methods

Taking its detection limit under consideration, we propose the use of this biosensor to analyze samples from aquatic environments prone to TEB contamination such as superficial waters and wastewaters. Additionally, this novel approach could be used to monitor TEB in drinking water, particularly water from environments close to agricultural fields. In these cases, the presence of TEB will lead to the upregulation of *ERG25*, consequently increasing the expression of the reporter gene yNluc and subsequent production of luciferase protein. This increase will be reflected in bioluminescence emission upon addition of the luciferase substrate. Absence of a bioluminescence signal indicates that no TEB is present or that TEB present in the sample in concentrations under 5 μg L^−1^, while a significative bioluminescence signal is indicative of TEB presence (Fig. 6). The results gathered in this study indicate that this new method can be an important complementary tool, namely in high throughput scenarios requiring screening of numerous samples, which can be performed by minimally skilled personnel in standard laboratories before chemical analysis by specialized services. In this manner, samples eliciting a positive signal can be further analyzed through conventional methods. Ultimately, this type of screening allows fast and inexpensive monitoring of TEB contamination in aquatic ecosystems, decreasing its economic cost and consequently enabling the implementation of semi-continuous environmental monitoring of TEB.

## Supplementary information

ESM 1 (PDF 365 kb)

## Data Availability

The data used to support the findings of this study are available from the corresponding author upon reasonable request.
