# Supplementary material for: Novel yeast-based biosensor for environmental monitoring of tebuconazole
Source: Appl Microbiol Biotechnol. 2024 Jan 3;108:10. doi: 10.1007/s00253-023-12944-z (PMC10764535; doi:10.1007/s00253-023-12944-z)
Supplement: Supplementary file 1 [file 253_2023_12944_MOESM1_ESM.pdf]

Supplementary Material

Novel yeast-based biosensor for environmental monitoring of tebuconazole

Filipa Mendes[1], Eduarda Miranda[1], Leslie Amaral[1], Carla Carvalho[1,2], Bruno B. Castro[1,3], Maria João Sousa[1,3], Susana R. Chaves[1*]

[1] CBMA – Centre of Molecular and Environmental Biology / ARNET – Aquatic Research Network, Department of Biology, School of Sciences, University of Minho, 4710-057 Braga, Portugal

[2] Present address: Centre for Environmental and Marine Studies (CESAM), Department of Biology, University of Aveiro, Aveiro, Portugal

[3] Institute of Science and Innovation for Bio-Sustainability (IB-S), School of Sciences, University of Minho, 4710-057 Braga, Portugal

*Corresponding author: Susana R. Chaves; E-mail address: suchaves@bio.uminho.pt

Table S1 List of *S. cerevisiae* strains and plasmids used in this study

**Strain**	**Genotype**	**Source**
BY4741	MAT a his3Δ1; leu2Δ0; met15Δ0; ura3Δ0	Euroscarf
BY4741 p*ERG3*pr-yNluc	BY4741 harboring pRS426-ERG25pr-yNluc	This Study
BY4741 p*ERG6*pr-yNluc	BY4741 harboring pRS426-ERG25pr-yNluc	This Study
BY4741 p*ERG10*pr-yNluc	BY4741 harboring pRS426-ERG25pr-yNluc	This Study
BY4741 p*ERG11*pr-yNluc	BY4741 harboring pRS426-ERG25pr-yNluc	This Study
BY4741 p*ERG25*pr-yNluc	BY4741 harboring pRS426-ERG25pr-yNluc	This Study
**Plasmid**	**Description**	**Source**

p*ERG3*pr-yNluc	*ERG3* promoter fused with yNLuc	This Study
p*ERG6*pr-yNluc	*ERG6* promoter fused with yNLuc	This Study
p*ERG11*pr-yNluc	*ERG11* promoter fused with yNLuc	This Study
p*ERG25*pr-yNluc	*ERG25* promoter fused with yNLuc	This Study

Table S2 List of primers used in this study

	Forward Primer 5'–3'	Reverse Primer 5'–3'
*ERG3*pr	CACCATCGTCGTCCTCCTGTTC	GATAATAAGAAAAATATTCGTCTAGATTTGA
*ERG6*pr	TGCTCGCTATCCTCGCCATC	AACAAGAATAAAATAATAATATAGTAGGCAGCATAAG
*ERG11*pr	CTTGTTCTCTCTCGCTTCCTACGTT	GAATAGAAACAGAACAAACGAGTAATACAAGG
*ERG25*pr	CTTTCATCCGTCTCGTTTATCATAA	GTACAGCCATAAAAAAAAGAGGAAAAGCTT
yNluc	ATGGTTTTTACTTTAGAAGATTTTGTTGG	GCGTTTATGTGAACGTATTTTAGCTTAA

Table S3 Mutator phenotype of *mlh1*Δ cells exposed to tebuconazole (TEB)

Time (h) / Treatments	*hom3-10* ($\times 10^{-5}$)[a]		
	**24**	**48**	**72**
**Control**	3.06 ± 2.3	1.56 ± 0.9	1.56 ± 1.5
**500 µg L^{-1}**	1.21 ± 0.5	0.962 ± 0.6	1.16 ± 1.0
**50 000 µg L^{-1}**	1.03 ± 0.2	1.21 ± 0.9	1.37 ± 1.4

[a] Median frequency of seven cultures. Values not statistically significant (Mann–Whitney test).

To determine whether tebuconazole (TEB) is genotoxic and can induce genomic instability, fluctuation analysis (Lea and Coulson, 1949) was used. The reporter strain RDKY3615 Δ*mlh1* carries a specific mutation that facilitates quantification of mutation rates, *hom3-10*. The *HOM3* gene encodes aspartate kinase (L-aspartate 4-P-transferase), a cytoplasmic enzyme necessary for threonine biosynthesis (Farfán et al., 1999). Thus, if yeast *hom3-10* mutants are grown on a medium lacking threonine, cells will die unless they revert the mutation. The strain also carrying a mutation in the *MLH1* gene, which plays a role in DNA repair, was used to facilitate measurements as it displays increased basal mutation frequencies (Argueso et al., 2003). Individual RDKY3615 *mlh1*Δ colonies were grown overnight in SC-Glucose medium with L-proline, diluted in fresh medium to

$OD_{640\ nm}$ = 0.1 and grown for an additional 3 h. Afterwards, cells were exposed to 0, 500 or 50 000 µg L^{-1} TEB. After 24, 48 and/or 72 h of treatment, cells were collected, washed, diluted in water, and appropriate dilutions plated on SC-Glucose medium (complete and lacking threonine). After 2 days at 30 °C, colonies were counted, and mutation frequencies calculated as described (Shell et al., 2007).

Table S4 Environmental water sample locations and characteristics. For each sample collected, characteristics such as temperature, dissolved oxygen (% saturation), total phosphorus, electrical conductivity (EC), total dissolved solids (TDS) and pH were determined

Sample	Name	Type	River Basin	Latitude	Longitude	Temperature (˚C)	Dissolved Oxygen (% sat.)	Total Phosphorus (mg P/L)	EC (µS/cm)	TDS (mg/L)	pH
**A**	Ribeira do Tojal	River	Cávado	41,684667	-8,415025	18,4	90	0,018	138	87	6,48
**B**	Rio Vez	River	Lima	41,880877	-8,426432	20,0	105	0,067	209	138	6,86
**D**	Refoios do Lima	River	Lima	41,781506	-8,539807	23,1	112	0,063	163	105	7,06
**F**	Caniçada	Reservoir	Cávado	41,676042	-8,180388	23,2	108	0,025	42	27	7,00
**G**	Oliveira	River	Ave	41,58626	-8,22512	17,7	102	0,012	137	86	6,82
**H**	Andorinhas	Reservoir	Ave	41,571226	-8,179073	20,4	93	0,015	109	70	6,64
**I**	Este (INL)	River	Ave	41,554336	-8,398938	19,2	86	0,122	240	156	6,97
**J**	Ucha	River	Cávado	41,569742	-8,526438	18,8	90	0,070	178	115	6,84

Table S5 Limit of detection (LoD) and concentration of fungicides (carbendazim, cooper, dimetomorph, metalaxyl and TEB) detected at environmental water samples analyzed by analytical methods. Samples where fungicide concentration is below the detection limit are identify as ND (not detected)

		Sample							
**Fungicide (μg L^{-1})**	**LoD**	**A**	**B**	**D**	**F**	**G**	**H**	**I**	**J**
**Carbendazim**	**0.002**	ND	ND	ND	ND	ND	ND	0.015	0.003
**Copper**	**0.15**	2.16	1.16	0.92	1.71	1.09	0.91	3.19	6.03
**Dimetomorph**	**0.002**	0.028	ND	ND	ND	ND	ND	0.005	0.006
**Metalaxyl**	**0.002**	0.043	0.006	ND	ND	0,005	0.003	0.003	0.006
**Tebuconazole (TEB)**	**0.005**	0.014	ND	ND	ND	ND	ND	ND	ND

Supplementary References

Argueso, J.L., Kijas, A.W., Sarin, S., Heck, J., Waase, M., Alani, E., 2003. Systematic Mutagenesis of the *Saccharomyces cerevisiae* MLH1Gene Reveals Distinct Roles for Mlh1p in Meiotic Crossing Over and in Vegetative and Meiotic Mismatch Repair. Mol. Cell. Biol. 23, 873–886.

Farfán, M.-J., Aparicio, L., Calderón, I.L., 1999. Threonine overproduction in yeast strains carrying the HOM3-R2 mutant allele under the control of different inducible promoters. Appl. Environ. Microbiol. 65, 110–116.

Lea, D.E., Coulson, C.A., 1949. The distribution of the numbers of mutants in bacterial populations. J. Genet. 49, 264–285.

Shell, S.S., Putnam, C.D., Kolodner, R.D., 2007. The N Terminus of *Saccharomyces cerevisiae* Msh6 Is an Unstructured Tether to PCNA. Mol. Cell 26, 565–578.
